# Effect of reperfusion therapy on QTd and QTcd in patients with acute STEMI

**DOI:** 10.1186/cc9422

**Published:** 2011-03-11

**Authors:** D Ragab, H Elghawaby, M Eldesouky, T Elsayed

**Affiliations:** 1 Cairo University, Cairo, Egypt

## Introduction

Acute ischemia alters action potentials and affects myocardial repolarization. Dispersion of repolarization is arrhythmogenic. QT dispersion has been suggested to give information about the heterogeneity of myocardial repolarization.

## Methods

Our study included 60 patients presented with acute STEMI, the study populations were divided into two groups: Group I: 30 patients who underwent primary PCI. Group II: 15 patients who received streptokinase. Group III: 15 patients who did not receive reperfusion therapy. QTd and QTcd were measured and compared in the three groups on admission, after 24 hours and after 5 days.

## Results

QTd and QTcd were significantly higher in patients with anterior compared with inferior MI (79.16 ± 25.67 ms vs. 62 ± 18.17 ms, *P *= 0.004 regarding QTd and 91.95 ± 28.76 ms vs. 68.33 ± 23.52 ms, *P *< 0.001 regarding QTcd). After 24 hours, QTd and QTcd were significantly lower in group I than groups II and III (34.33 ± 13.56 ms vs. 48 ± 18.2 ms vs. 66 ± 24.43 ms respectively, *P *< 0.05 as regards QTd and 39.33 ± 11.72 ms vs. 56 ± 23.84 ms vs. 74.60 ± 26.7 ms respectively, *P *< 0.05 as regards QTcd). On the 5th day reduction in QTd and QTcd was statistically significantly lower in group I than groups II and III (23 ± 9.52 ms vs. 45.33 ± 15.97 ms vs. 58.66 ± 23.25 ms respectively, *P *< 0.05 for QTd and 26 ± 11.63 ms vs. 52.66 ± 21.2 ms vs. 60.66 ± 23.25 ms respectively, *P *< 0.05 for QTcd). QT and QTcd on admission were higher in patients who developed ventricular arrhythmias than patients who did not (90 ± 11.55 ms vs. 70 ± 24.54 ms; *P *= 0.05 regarding QTd and 110 ± 8.61 ms vs. 80.53 ± 28.78 ms with *P *= 0.028 regarding QTcd). Patients with early peaking of enzymes had more reduction in QTd and QTcd early after reperfusion (43.2 ± 11.44 vs. 60.5 ± 13.16, *P *< 0.001 regarding QTd and 49.60 ± 15.93 vs. 68.5 ± 17.55, *P *< 0.001 regarding QTcd).

## Conclusions

QTd is higher in patients with acute MI (AMI) who developed ventricular arrhythmias. So QTd and QTcd on admission may be a helpful parameter that can detect patients with AMI who are at risk for development of ventricular arrhythmias. Reperfusion therapy with primary PCI or thrombolytic agents reduces QTd and QTcd in patients with AMI, however; QTd and QTcd are shorter with primary PCI compared with thrombolytic therapy.

